# Degenerative Spondylolisthesis Is Associated with Low Spinal Bone Density: A Comparative Study between Spinal Stenosis and Degenerative Spondylolisthesis

**DOI:** 10.1155/2013/123847

**Published:** 2013-08-19

**Authors:** Thomas Andersen, Finn B. Christensen, Bente L. Langdahl, Carsten Ernst, Søren Fruensgaard, Jørgen Østergaard, Jens Langer Andersen, Sten Rasmussen, Bent Niedermann, Kristian Høy, Peter Helmig, Randi Holm, Niels Egund, Cody Bünger

**Affiliations:** ^1^Orthopaedic Department, Spine Section, Aarhus University Hospital, Nørrebrogade 44, 8000 Aarhus C, Denmark; ^2^Department of Endocrinology and Metabolism, Aarhus University Hospital, Denmark; ^3^Orthopaedic Department, Esbjerg County Hospital, Denmark; ^4^Orthopaedic Department, Holstebro County Hospital, Denmark; ^5^Orthopaedic Department, Viborg County Hospital, Denmark; ^6^Orthopaedic Department, Vejle County Hospital, Denmark; ^7^Department of Radiology, Aarhus University Hospital, Denmark

## Abstract

Spinal stenosis and degenerative spondylolisthesis share many symptoms and the same treatment, but their causes remain unclear. Bone mineral density has been suggested to play a role. The aim of this study was to investigate differences in spinal bone density between spinal stenosis and degenerative spondylolisthesis patients. 81 patients older than 60 years, who underwent DXA-scanning of their lumbar spine one year after a lumbar spinal fusion procedure, were included. Radiographs were assessed for disc height, vertebral wedging, and osteophytosis. Pain was assessed using the Low Back Pain Rating Scale pain index. *T*-score of the lumbar spine was significantly lower among degenerative spondylolisthesis patients compared with spinal stenosis patients (−1.52 versus −0.52, *P* = 0.04). Thirty-nine percent of degenerative spondylolisthesis patients were classified as osteoporotic and further 30% osteopenic compared to only 9% of spinal stenosis patients being osteoporotic and 30% osteopenic (*P* = 0.01). Pain levels tended to increase with poorer bone status (*P* = 0.06). Patients treated surgically for symptomatic degenerative spondylolisthesis have much lower bone mass than patients of similar age treated surgically for spinal stenosis. Low BMD might play a role in the development of the degenerative spondylolisthesis, further studies are needed to clarify this.

## 1. Introduction

Spinal stenosis and degenerative lumbar spondylolisthesis are common conditions in the aging spine. The degenerative spondylolisthesis can subsequently lead to severe spinal stenosis due to the progressive slip. Surgery for these two conditions, decompression and in many cases fusion, has shown increasing rates during the last decade [1]. But, although these two conditions share many symptoms and the same treatment, the causes of the two different disease entities remain unclear. Recent work focuses on the genetic component of degenerative disc disease in its widest sense, which seems large [2], but so far genetic differences between these two groups of patients have not been reported. Degenerative spondylolisthesis has been reported to be 4-5 times more common in women than in men, although a recent report states that the presence in men might be underestimated [3]. Hormonal influences, pregnancy, pelvic morphology, and facet joint orientation have been suggested as causes [4–8]. Very few of the studies have looked beyond morphology of the spine when looking for differences between degenerative spondylolisthesis and spinal stenosis. One study has shown different amounts of matrix metalloproteinases in ligamentum flavum in these two patient groups, suggesting a biochemical pathway for increased collagen laxity, which subsequently could lead to the slip [9]. Bone mineral density has been shown to be independently associated with degenerative disc disease [10], and one study, on bone mineral density of the lumbar spine in elderly women, with degenerative spondylolisthesis, has suggested different pathomechanisms on different levels. But in general the role of bone mineral density in lumbar degenerative disease is not very well understood.

We utilized data from a surgical cohort of elderly patients undergoing fusion surgery for degenerative disc disease, spinal stenosis, or degenerative spondylolisthesis in order to investigate differences in spinal bone mass between these diagnostic groups.

## 2. Materials and Methods

### 2.1. Patients

The patient cohort in this study is taken from a multicenter randomized trial on the effect of direct current (DC) electrical stimulation in adjunct to uninstrumented spinal fusion [11, 12]. All patients underwent uninstrumented posterolateral spinal fusion using fresh frozen allograft and were braced postoperatively for 3-4 months. This study includes 81 patients with a preoperative diagnosis of either degenerative disc disease (DDD), spinal stenosis, or degenerative spondylolisthesis (DS), who underwent spinal bone densitometry at 1 year postoperatively. Patient demographics are seen in Table 1. Five patients had a history of a prior decompressive procedure and 9 patients had previously had lumbar discectomy, the remaining patients had not been operated on in their lumbar spine.

### 2.2. Bone Densitometry

**Figure 1:**
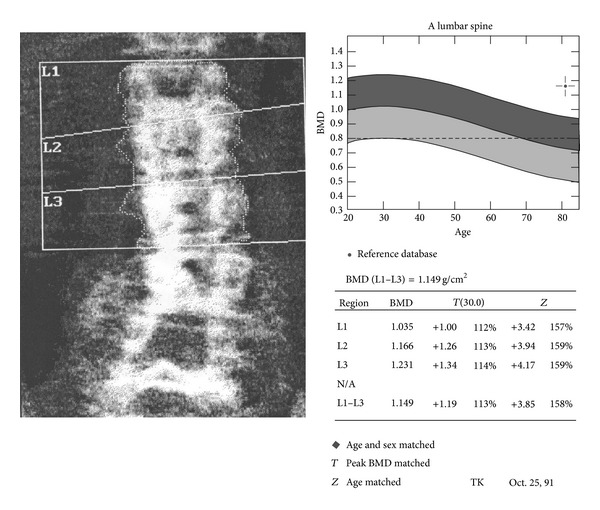
Main results from the lumbar AP scanning. The figure illustrates the inclusion of only those vertebrae not part of the fusion in the calculation of the overall bone mass.

Bone densitometry of the lumbar spine was performed 1 year after the fusion surgery. Bone mineral density (BMD, g/cm^2^) and bone mineral content (BMC, g) were measured by dual energy X-ray absorptiometry (DXA) using a Hologic QDR-2000 densitometer (Hologic Inc., Waltham, MA, USA). Lumbar spine BMD and BMC were assessed using a standard anteroposterior L1–L4 scanning including only the lumbar vertebrae above the fused levels, for example, L1–L3 in a patient with an L4-S1 fusion (Figure 1). *T*- and *Z*-scores were calculated using the scanner software and reference values. The *T*-score is the number of standard deviations above or below the mean for a healthy 30-year-old adult of the same sex and ethnicity as the patient, whereas the *Z*-score is the number of standard deviations above or below the mean for the patient's age, sex, and ethnicity. Values were calculated for each vertebra as well as an overall mean of the included vertebrae; the latter was used as a measure of the patients overall bone status.

### 2.3. Radiographs

The latest available lateral spine radiographs were used for measurements. Only standing pictures, taken without brace, were accepted for measurements. Four measurements were performed: Disc height, vertebral wedging, osteophyte score, and lumbar lordosis. disc height was measured using the Farfan method allowing for correction of differences due to magnification [13]. Vertebral wedging was measured as the anterior vertebral height relative to the posterior vertebral height; thus the lower a value below 1 the more the vertebra has collapsed anteriorly. The degree of osteophytosis was assessed at each disc level above the fusion. It was scored from 0 (no osteophytes) to 4 (osteophytes fusing the segment) using the score proposed by Nathan [14]. If a score between two categories was deemed appropriate, a middle value was allowed to be assigned (e.g., 2.5 for a score between category 2 and category 3). Lumbar lordosis was measured, as suggested by Wiltse and Winter [15], on the lateral radiographs as the angle between a line drawn across the top of the body of the first lumbar vertebra and one drawn across the top of the body of the fifth lumbar vertebra.

### 2.4. Pain and Disability Assessment

Pain was assessed using the pain assessment index from the Low Back Pain Rating Scale (LBPRS) [16]. It is measured using 11-box numerical rating scales ranging from 0 representing no pain to 10 representing worst possible pain. It comprises three scales for back and leg pain separately (pain now, worst, and average pain last 14 days). Each response scale is added giving a scale ranging from 0 to 60. Functional outcome was assessed using the Dallas Pain Questionnaire (DPQ) [17], which assesses the functional impact of chronic spinal pain in four categories: daily activities, work-leisure activities, anxiety and depression, and social concerns. A high score indicates a high influence of back pain on the daily life of the patient and thus a poor function. Scores obtained from the clinical follow-up coming closest to the date for DXA-scanning were utilised for this measure.

### 2.5. Statistics

Between groups' comparisons were done using Kruskal-Wallis tests with adjustment for ties, Mann-Whitney rank sum test, or chi-square test, whichever was appropriate. Test for trends across groups was tested using nonparametric trend test. Two-way analysis was done using two-way Anova. Linear regression was done as standard ordinary least-squares regression. Level of significance was set to 0.05 (two-tailed testing). Intercooled Stata version 12 for Windows was the software used for all analysis.

## 3. Results

Seventeen percent (14/81) of the patients were classified as osteoporotic, with a further 26% (21/81) being osteopenic. Stratifying for gender, the numbers were 6/29 (21%) osteoporotic and 6/29 (21%) osteopenic among the males and 8/52 (15%) osteoporotic and 15/52 (29%) osteopenic among the females (*P* = 0.67). On the average, however, the patients had a higher bone mass as compared to their age- and sex-matched groups, as illustrated by the overall *Z*-score (Table 2). Bone status was associated with pain history as 86% (12) of the osteoporotic patients had a preoperative pain history of more than two years compared to 67% (14) of the osteopenic patients and only 59% (27) of those patients with normal bone status. In general, bone status declined from the lower lumbar vertebrae to the upper lumbar vertebrae (Table 2).

Lower BMD and *T*-scores at all vertebrae were seen in patients diagnosed with degenerative spondylolisthesis or degenerative disc disease compared to patients with spinal stenosis, and this resulted in a significantly higher proportion of osteoporotic patients in the former categories (Table 2). There was no difference in disc height and vertebral wedging between the diagnostic groups, but a tendency towards a lower osteophyte score in degenerative spondylolisthesis patients was seen (Table 2). The difference in bone density parameters was not due to the larger proportion of women among the degenerative spondylolisthesis patients as BMD; *T*-score and *Z*-score were lower in these patients in both sexes (data not shown). Linear regression confirmed that a diagnosis of degenerative spondylolisthesis was associated with lower BMD after controlling for other contributory factors, although it did not reach statistical significance (Table 3).

**Figure 2:**
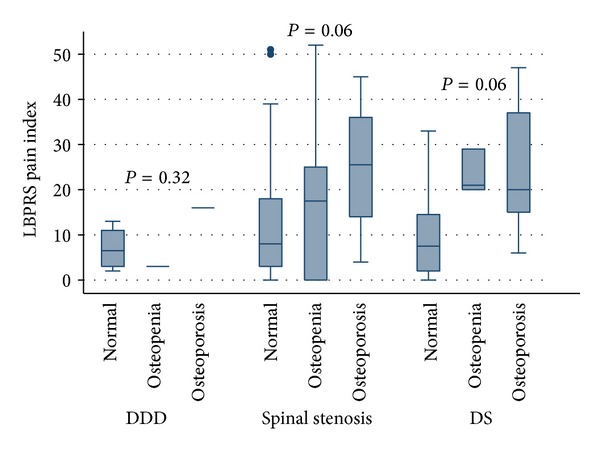
Boxplot of the Low Back Pain Rating Scale pain index at follow-up closest to DXA-scanning. *P* values are nonparametric test for trend across group. Two-way Anova results in no effect of diagnosis (*P* = 0.42), but significant effect of bone status (*P* = 0.02). DDD: Degenerative disc disease. DS: Degenerative spondylolisthesis.

**Figure 3:**
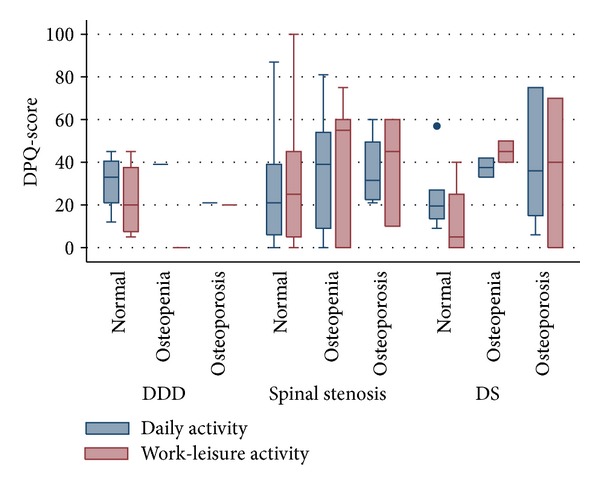
The two activity- and function-related scores from the DPQ according to diagnosis and bone status. There is no significant trend across the groups in any of the groups (*P*  values > 0.10) DDD: Degenerative disc disease. DS: Degenerative spondylolisthesis.

Osteoporotic patients had significantly higher pain levels one year after their lumbar fusion procedure compared to patients with normal bone status (Figure 2). The difference in the LBPRS pain index was due to higher scores in both back and leg pain. Their functional status, as assessed by the DPQ, was however not affected (Figure 3). The same was true for the preoperative function (data not shown), whereas preoperative pain levels were higher in patients with low bone mass (Mean (sd)): 35 (15) versus 42 (16) versus 42 (9), *P* = 0.15 for normal, osteopenic, and osteoporotic patients, respectively.

## 4. Discussion

We found the lowest BMD-values and *T*-scores in patients surgically treated for degenerative spondylolisthesis and the highest BMD-values and *T*-scores in patients operated on because of spinal stenosis. The latter group also had the smallest fraction of osteoporotic patients. When measured in the AP-spine, BMD has been shown to be elevated, as compared to measures taken at peripheral skeletal sites, when degenerative changes (facet arthrosis, osteophytes etc.) are present [18–20]. In accordance with this, we did find the highest degree of osteophytosis in this patient group but not in a uniform manner and not to an extent that can explain the differences between these two groups. Other studies have also suggested a connection between high BMD and development of osteoarthritis [20, 21]. Regarding the cause of degenerative spondylolisthesis, several studies have investigated the morphology of the facet joints as a possible cause of the degenerative slip and found that the angulation was associated with slip and changed through the decades of life, thus explaining the fact that the degenerative slip first occurs in the later part of life [5, 22]. Love et al. found angles of the facet joints to be significantly different in patients (both male and female) with degenerative spondylolisthesis compared to those without. They speculated that the cause could be a generalized osteoarthritic condition, which could explain the occurrence of degenerative spondylolisthesis due to arthritic remodelling [4]. One study has investigated the relation between BMD, both spinal and hip, and facet joint orientation and found no difference in facet joint orientation between osteoporotic, osteopenic, and normal patients [23]. That study however excluded patients with spondylolisthesis, so whether the findings hold true for degenerative spondylolisthesis patients remains unknown. Our study did not allow for facet joint evaluation, as only lateral radiographs were utilized. And, although the cross-sectional design of the present study does not allow for conclusions regarding causality, it might be speculated that sagittally oriented facet joints is a prerequisite for development of degenerative spondylolisthesis, but that this development only occurs in patients with low BMD, perhaps because they are unable to generate a remodelling response that will cause the formation of osteophytes which subsequently will stabilize the olisthesis and prevent it developing into a clinical significant slip. Contradictory to this, Vogt et al. reported higher BMD in patients with anterolisthesis compared to patients without olisthesis at some levels [24]. This was however not the case for the L4-L5 level, where significant differences could not be observed. Furthermore, their population was taken from a study investigating osteoporotic fractures and included only women. As our population included both genders and is characterised by the fact that they elicit spinal degenerative changes significant enough to undergo surgery, for the majority due to stenosis symptoms, this makes it difficult to compare the two populations. In a surgical patient population, the SPORT trial showed significant differences between degenerative spondylolisthesis and spinal stenosis patients, with a much larger proportion of the latter having multilevel involvement [25]. Another factor not investigated in the current study is the influence of sagittal balance. Recently, differences between patients with or without degenerative spondylolisthesis have been shown [26], but unfortunately the radiographs available for the present study did not allow for measurements of the fundamental pelvic parameters as they did not include the hips. To our knowledge, no study has yet investigated associations between bone mineral density of the spine and sagittal balance parameters.

In general, BMD of the single vertebrae has been shown to vary within the lumbar spine, but with a decline going from L5 to L1 being the most occurring phenomenon [27]. We observed similar trends in this study. This is however unlikely to be the explanation to the difference observed between patients with spinal stenosis and those with degenerative spondylolisthesis. As there were more multilevel fusions in the latter group, this would mean that fewer of their overall BMD measurements would include lower vertebra and would lead to a falsely low BMD, thus reducing the difference observed between the two groups.

Also the group of stenosis patients had the largest proportion of smokers. Smoking has been associated with low BMD [28] and this would therefore also tend to reduce the difference observed between the two populations. Another variable associated with a slower decline in BMD in older age is activity [28]. This might play a role as we could show that osteopenia and osteoporosis were associated with higher pain levels; thus, osteoporotic patients might have a lower activity level and therefore more rapid decline in bone mass and, hence, there were more osteoporotic patients in the degenerative spondylolisthesis group; this could be one explanation to our finding. The capacity to do activity, in form of self-reported walking distance, was however equal between the two groups, both preoperatively and at follow-up. The same was true for the scores assessing functional impairment.

Studies have shown BMD of vertebrae above a fusion to elicit an initial decline after lumbar spine surgery, with a subsequent return to values above baseline as early as after one year [29–32]. Chin et al. reported somewhat poorer values obtained from a preoperative DXA scanning of patients above 50 years undergoing spine surgery but in an Asian population [33]. Thus, it cannot be completely ruled out that the BMD-measurements in this study slightly overestimates the preoperative status, but it seems unlikely that any effect of the surgery, on bone status of the adjacent vertebrae, should vary between the different diagnostic entities.

We found low bone mass to be associated with more pain. In a cross-sectional study, Manabe et al. could demonstrate that a high BMD increased the risk of low back pain in women aged 45 to 64 years with an OR of 1.40. This association was, however, not present in the women aged 65 and above [34]. Investigating chronic low back pain patients undergoing rehabilitation, Gaber et al. failed to demonstrate any correlation between BMD and pain or disability, as assessed with the Oswestry Disability Questionnaire [35]. Likewise, Nicholson et al. could not demonstrate any association between history of back pain and BMD or *Z*-score [36]. One explanation to our finding could be a higher degree of inactivity in the patients with more severe pain, leading to a larger bone loss or a reduced increase in BMD after the operation. Another possibility could be a higher proportion of nonunion patients among the osteoporotic patients, resulting in higher pain levels, but against this speaks the fact that also the preoperative pain levels were higher among the osteoporotic/osteopenic patients.

In conclusion, patients treated surgically for symptomatic degenerative spondylolisthesis have much lower bone mass than patients of similar age treated surgically for spinal stenosis. Low BMD might play a role in the development of the degenerative spondylolisthesis, further studies are needed to clarify this. 

## Figures and Tables

**Table 1:** Patient demographics.

	DDD	Spinal stenosis	Degenerative spondylolisthesis	*P* value
Gender (m/f)	2 (29%)/5 (71%)	23 (41%)/33 (59%)	4 (22%)/14 (78%)	0.32^1^
Age at DEXA	66 (4)	72 (6)	73 (6)	0.034^2^
Pain history				0.15
<1 year	0 (0%)	10 (18%)	1 (6%)	
1-2 years	1 (14%)	9 (16%)	7 (39%)	
>2 years	6 (86%)	37 (66%)	10 (56%)	
Preoperative smoking				0.19
Nonsmoker	4 (57%)	34 (61%)	15 (83%)	
Smoker	3 (43%)	22 (39%)	3 (17%)	
Number of fused levels				0.002^3^
1	5 (71%)	10 (18%)	9 (50%)	
2	1 (14%)	30 (54%)	8 (44%)	
3	0 (0%)	15 (27%)	1 (6%)	
4	1 (14%)	1 (2%)	0 (0%)	
Additional neural decompression				<0.001^4^
None	6 (86%)	0 (0%)	0 (0%)	
Laminotomy	1 (14%)	11 (20%)	4 (22%)	
Laminectomy	0 (0%)	45 (80%)	14 (78%)	
Preoperative walking distance (median/iqr)	500 (400)	200 (500)	200 (400)	0.81^5^
Follow-up walking distance (median/iqr)	350 (4900)	2000 (8999)	1500 (1000)	0.24^6^

Demographic parameters in the three diagnostic groups. DDD: degenerative disc disease.

When performing two-group comparison (spinal stenosis versus degenerative spondylolisthesis): ^1^ *P* = 0.15, ^2^ *P* = 0.82, ^3^ *P* = 0.03, ^4^ *P* = 0.81, ^5^ *P* = 0.63, ^6^ *P* = 0.26.

Values are mean (sd) or *n* (%).

**Table 2:** Bone densitometry and radiographic parameters.

	DDD	Spinal stenosis	Degenerative spondylolisthesis	*P* value
BMC (g)				
L4	14.29 (4.21)	16.70 (2.59)	NA	0.35
L3	14.42 (5.99)	18.48 (4.90)	13.42 (3.41)	0.02
L2	12.65 (4.16)	15.20 (4.70)	11.45 (2.78)	0.01
L1	11.96 (4.39)	12.57 (3.91)	10.20 (2.94)	0.08
BMD (g/cm^2^)				
L4	0.812 (0.141)	1.059 (0.347)	NA	0.35
L3	0.847 (0.290)	1.122 (0.215)	0.942 (0.175)	0.04
L2	0.874 (0.254)	0.993 (0.210)	0.873 (0.206)	0.10
L1	0.851 (0.232)	0.884 (0.198)	0.794 (0.175)	0.22
*T*-score				
L4	−2.90 (1.32)	−0.65 (3.34)	NA	0.35
L3	−2.22 (2.68)	0.26 (1.95)	−1.33 (1.58)	0.06
L2	−1.60 (2.41)	−0.56 (1.85)	−1.52 (1.95)	0.17
L1	−0.89 (2.19)	−0.68 (1.71)	−1.36 (1.62)	0.33
*Z*-score				
L4	−1.60 (1.83)	0.96 (4.02)	NA	0.35
L3	−0.80 (3.17)	1.78 (2.08)	0.79 (1.76)	0.13
L2	−0.07 (2.88)	1.10 (1.94)	0.49 (2.26)	0.42
L1	0.51 (2.48)	0.86 (1.76)	0.43 (1.86)	0.73
Overall				
BMC (g)	41.27 (21.27)	31.10 (16.49)	28.58 (12.12)	0.38
BMD (g/cm^2^)	0.884 (0.219)	0.954 (0.205)	0.855 (0.179)	0.11
*T*-score	−1.32 (2.28)	−0.52 (1.75)	−1.42 (1.70)	0.10^#^
*Z*-score	0.20 (2.68)	1.09 (1.81)	0.51 (2.20)	0.43*
Bone status				0.05
Normal	4 (57%)	34 (61%)	8 (44%)	
Osteopenia	1 (14%)	17 (30%)	3 (17%)	
Osteoporosis	2 (29%)	5 (9%)	7 (39%)	
Disc height				
L4-L5	0.25 (0.03)	0.25 (0.02)	NA	0.64
L3-L4	0.20 (0.05)	0.21 (0.06)	0.22 (0.06)	0.62
L2-L3	0.15 (0.04)	0.18 (0.05)	0.18 (0.05)	0.43
L1-L2	0.12 (0.05)	0.16 (0.04)	0.13 (0.06)	0.23
Osteophyte score				
L4-L5	0.75 (0.65)	2.00 (0.00)	NA	0.06
L3-L4	1.20 (0.57)	1.38 (0.82)	0.67 (0.43)	0.10
L2-L3	1.38 (0.48)	1.42 (0.86)	0.89 (0.53)	0.07
L1-L2	1.50 (0.58)	1.27 (0.83)	1.07 (0.62)	0.47
Vertebral wedging				
L4	1.05 (0.06)	0.99 (0.06)	NA	0.35
L3	0.96 (0.06)	1.01 (0.08)	1.01 (0.05)	0.27
L2	0.90 (0.02)	0.93 (0.08)	0.95 (0.10)	0.48
L1	0.99 (0.26)	0.90 (0.10)	0.93 (0.09)	0.51
Lordosis	19° (18°)	24° (15°)	30° (11°)	0.21

Difference in bone densitometry and radiographic parameters between the three diagnostic groups.

when performing two-group comparison of spinal stenosis and degenerative spondylolisthesis ^#^ *P* = 0.04, *P = 0.26.

Values are mean (sd) or *n* (%). NA: not applicable.

**Table 3:** Linear regression with BMD of the unfused spine as dependent variable.

Factor	Coefficient	SE	95% CI	*P* value
Age	−0.001	0.004	−0.009–0.007	0.769
Degenerative spondylolisthesis	−0.093	0.055	−0.164–0.057	0.340
Smoking	−0.084	0.051	−0.196–0.009	0.074
Walking distance (km)	0.022	0.010	0.000–0.043	0.035
Female gender	−0.107	0.049	−0.205–0.008	0.034
Osteophyte score (average)	0.035	0.031	−0.027–0.098	0.259
Constant	1.260	0.327	0.606–1.913	0.000

Analysis performed excluding patients with degenerative disc disease. Adjusted marginal mean BMD for spinal stenosis patients was 0.943 compared to 0.889 in degenerative spondylolisthesis patients (*P* = 0.336).

Adjusted *R* ^2^ = 0.147, SE: Standard error. CI: Confidence interval.
